# Association between depression and lung function in college students

**DOI:** 10.3389/fpubh.2023.1093935

**Published:** 2023-03-23

**Authors:** Cui Wang, Hongbo Chen, Shaomei Shang

**Affiliations:** ^1^School of Nursing, Peking University, Beijing, China; ^2^School of Public Health, Peking University, Beijing, China

**Keywords:** depression, lung function, college students, peak expiratory flow, forced vital capacity, forced expiratory volume in 1s

## Abstract

**Background:**

Depression is positively associated with lung dysfunction in middle-aged and older adults, but the correlation between depression and lung dysfunction in healthy young adults has not been well researched.

**Methods:**

This cross-sectional study used a spirometer to evaluate the lung function of 352 college students (mean age: 24.1 years). The spirometry measurements included the peak expiratory flow (PEF), predicted percentage of the peak expiratory flow (PEF pp), forced expiratory volume in 1 s (FEV1), predicted percentage of the FEV1 (FEV1 pp), forced vital capacity (FVC), predicted percentage of the FVC (FVC pp), FEV1/FVC ratio and the predicted percentage of the FEV1/FVC ratio (FEV1/FVC pp). A validated Chinese version of the 20-item Zung Self-rating Depression Scale (SDS) was used to assess the severity of depression among young adults, with scores of ≥ 40 and ≥ 45 points indicating mild and moderate-to-severe depression, respectively. The Kruskal-Wallis tests were used to analyze the continuous variables, to estimate differences in lung function among the different levels of depression. Chi-square tests or Fisher’s exact tests were used to analyze the categorical variables, to estimate differences in characteristics among the different levels of depression. Several multiple logistic regression models were used to examine the associations between participants’ level of depression and each of the variables measuring lung function.

**Results:**

Mild and moderate-to-severe depression were observed in 9.9 and 7.4% of the students, respectively. In particular, mild depression was associated with reduced FEV1 in both unadjusted (OR = 1.498, *p* = 0.003) and adjusted models (OR = 1.290, *p* = 0.018; OR = 1.199, *p* = 0.044). On the other hand, moderate-to-severe depression was significantly but negatively related to FEV1 in both unadjusted (OR = 3.546, *p* = 0.005) and adjusted models (OR = 3.137, *p* = 0.020; OR = 2.980, *p* = 0.048). Furthermore, the unadjusted model indicated that mild depression was associated with a higher risk of a lower PEF (OR = 3.546, *p* = 0.008).

**Conclusion:**

Severe depression is an independent predictor of decreased FEV1 among Chinese college students.

## 1. Introduction

Decreased lung function can have deleterious effects on health outcomes and quality of life. Several known risk factors for low lung function include exposure to tobacco smoke and lack of physical activity, among others (1, 2). From a clinical and public health perspective, the identification of risk factors that negatively influence lung function is vital to the early detection of morbidity.

A previous study has shown that depression is closely linked to chronic lung diseases (3). According to prior report, 10%–42% of patients with stable chronic obstructive pulmonary disease (COPD) have depression, whereas those with acute COPD exacerbation have depression rates ranging from 10% to 86% of patients (3). Depression is related to a reduction in physical activity, and it likely contributes to a decline in lung function (4). Pro-inflammatory cytokines such as C-reactive protein and interleukin-6 seem to be involved in the relationship between depression and lung function (5). These findings indicate that depression may negatively influence pulmonary function. The Global Initiative for Chronic Obstructive Lung Disease has proposed conducting future studies to evaluate lung function in larger populations exposed to different risk factors, such as depression (6). Thus far, depression has been reported to be associated with lung dysfunction in individuals aged 55 years (3), but the correlation between the two variables among young adults has not been adequately researched.

## 2. Methods

### 2.1. Ethics statement

The participants provided their written informed consent to participate in the study. The Institutional Review Board of the Peking University Health Science Center reviewed and approved this study (IRB00001052-22073).

### 2.2. Study design and participants

College students from 20 Peking University Health Science Center schools were recruited using posters with information inviting students to participate in this cross-sectional study between July and September 2022. A self-administered survey questionnaire of health status was completed by each participant who subsequently underwent an assessment of lung function. The following exclusion criteria were applied: college students under 18 years of age, those who did not complete the questionnaire or the assessment of pulmonary function and those with COPD, asthma or tuberculosis.

### 2.3. Assessment of lung functions

The lung functions that were examined included the forced expiratory volume in 1 s (FEV1), predicted percentage of FEV1 (FEV1 pp), forced vital capacity (FVC), predicted percentage of FVC (FVC pp), peak expiratory flow (PEF), predicted percentage of PEF (PEF pp), FEV1/FVC ratio and the predicted percentage of FEV1/FVC ratio (FEV1/FVC pp). The measurements were obtained using a multi-functional spirometer (Suzhou, China). Participants were required to stand in a stationary position while holding the spirometer before performing a forced expiration. Each participant attempted the process three times; and the highest reading was recorded.

Since there are no established standards for defining normal values for lung function indicators such as FEV1, FVC, FEV1/FVC, PEF, and their corresponding percent predicted values among college students, we used median values to create two groups. For FEV1 and FEV1 pp, the cut-off values were 3.6 and 92.7%, respectively. For FVC and FVC pp, the cut-off values were 4.1 and 83.7%, respectively. For FEV1/FVC and FEV1/FVC pp, the cut-off values were 83.8 and 117.4%, respectively. For PEF and PEF pp, the cut-off values were 7.6 and 89.6%, respectively.

### 2.4. Assessment of the severity of depression

The severity of depression was assessed using a validated Chinese version of the Zung Self-rating Depression Scale (SDS), which contains 20 items, with ratings for each item having ranging from 1 to 4 points. The total possible score ranges from 20 to 80 pts., with higher scores indicating greater severity. Scores ≥ 40 pts. indicate mild depression and scores ≥ 45 pts. indicate moderate-to-severe depression (7). Based on their SDS scores, all participants were subsequently assigned to one of the following groups for the analysis of their results: normal (SDS score = 20–39 pts), mild depression (SDS score = 40–44) or moderate-to-severe depression (SDS score ≥ 45).

### 2.5. Relevant covariates

Based on previous research, we controlled for the effects of potential covariates such as sex, age, body mass index (BMI), physical activity, sleep quality, smoking and drinking status and anxiety (7–9).

Age and BMI were treated as continuous variables, and BMI was calculated as weight in kilograms (kg) divided by height in meters squared. Smoking status was divided into three categories and three corresponding groups of participants (current-, former- and non-smokers). Participants who were smoking at the time of the survey were classified as current smokers, those who smoked previously but had quit were classified as former smokers and participants who had never smoked were classified as non-smokers. Alcohol drinking was divided into three categories and three corresponding groups of participants (current-, former- and non-drinkers). Participants who were drinking alcohol at the time of the survey were classified as current drinkers, those who previously drank alcohol but had quit drinking it were classified as former drinkers and those who had never drank alcohol were classified as non-drinkers.

Participants’ physical activity was measured using the International Physical Activity Questionnaire Short Version (10). This questionnaire contained items that assessed how long and how often participants performed physical activity at one of the following levels of intensity: low physical activity (LPA = 3.3 metabolic equivalents [METs]), moderate physical activity (MPA = 4.0 METs) and vigorous physical activity (VPA = 8.0 METs). The activities could span various domains during a typical week of the participants’ lives (e.g., transport-related or work-related activities, domestic and gardening (yard) activities and leisure time). Based on the data collected and estimated energy expenditures (EE; expressed in METs·min/week), participants were assigned to one of the following groups, which reflected their level of physical activity: (1) High physical activity (HPA) – participants who met any one of the following criteria: participants who performed VPA for at least 3 days, with an EE of at least ≥ 1,500 METs·min/week, or those who performed any combination of activities at the three levels of intensity for seven or more days, with an EE of at least ≥3,000 METs·min/week; (2) Moderate physical activity (MPA) – participants who performed VPA activity for at least 20 min/day for 3 or more days or those who performed at MPA or LPA levels for at least 30 min/day for 5 or more days or those who performed any combination of LPA, MPA or VPA for 5 or more days, with an EE of at least ≥ 600 METs·min/week; or (3) Low physical activity (LPA) – participants without physical activity or those who reported some activity that was insufficient to meet at least the level of criteria for MPA.

Sleep quality was measured using the Pittsburgh Sleep Quality Index (11). This self-report questionnaire contains 19 items that assess sleep quality over the past month, and yields scores for seven components: daytime dysfunction, use of sleep medications, sleep disturbances, habitual sleep efficiency, duration of sleep, sleep latency and overall quality of sleep. The total scores of the components are summed to determine the overall Pittsburgh Sleep Quality Index. In this study, poor sleep quality during the previous month was indicated by higher scores.

Anxiety was assessed using the following five questions of the SDS: “Do you feel down-hearted and blue?,” “Do you have trouble sleeping at night?,” “Does your heart beat faster than usual?,” “Do you get tired for no reason?” and “Do you still enjoy the things you used to do?.” The total possible scores for the five items ranged from 5 to 20 pts., with higher scores indicating more severe anxiety. In this study, anxiety was defined as a score ≥ 6 (7).

### 2.6. Statistical analysis

IBM SPSS Statistics 24.0 software (IBM SPSS Inc., Chicago, IL, United States) was used for all the statistical analyses. Continuous variables are expressed as medians and 25th and 75th percentiles, and categorical variables are expressed as frequencies and percentages, *n* (%). Because the distributions of all indicators of lung function were skewed, Kruskal-Wallis tests were used to analyze the continuous variables, to estimate differences in lung function among the different levels of depression. Chi-square tests or Fisher’s exact tests were used to analyze the categorical variables, to estimate differences in characteristics among the different levels of depression.

The indicators of lung function and depression severity were used as dependent and independent variables, respectively. Since there are no established standards for defining normal values for lung function indicators (e.g., PEF, FEV1 and FVC), and their corresponding percent predicted values among college students, we used median values to create two groups. Several multiple logistic regression models were used to examine the associations between participants’ level of depression and each of the variables measuring lung function. No adjustments to model 1 were made for the analysis. Adjustments to model 2 were made for age (continuous variable) and sex (categorical variable). Model 3 was further adjusted for BMI (continuous variable), smoking status (categorical variable), drinking status (categorical variable), physical activity (categorical variable), sleep quality (continuous variable) and anxiety symptoms (categorical variable). A sensitivity analysis was performed to examine the reliability of the primary results. In the sensitivity analysis, the indicators of lung function and SDS scores were used as dependent and independent variables, respectively. The SDS score was used to divide participants into three groups in accordance with the cut-off values of the tertiles, and each lung function (e.g., PEF, FEV1 and FVC) was divided into two groups (cut-off values in median). Several multiple logistic regression models were then used to examine the associations between the SDS score tertiles and each of the variables measuring lung function. The results of all the two-tailed tests with p-values <0.05 were considered statistically significant.

## 3. Results

A total of 358 college students were recruited, and they all completed informed consent. We excluded 6 participants owing to missing data on depression (*n* = 3) and FEV1 values (*n* = 3). Therefore, data were analyzed for 352 participants with a mean age of 24.1 years (47.7% were males). As shown in Table 1, of the 352 participants, 35 (9.9%) had mild depression and 26 (7.4%) had moderate-to-severe depression. The level of physical activity among the latter group of participants, was significantly lower (*p* = 0.004) than that of the former group, as was the level of their quality of sleep (*p* < 0.001). However, participants with mild depression reported a higher BMI (*p* = 0.033) and more anxiety (*p* < 0.001) than their counterparts with moderate-to-severe depression (*p* < 0.001).

**Table 1:** Baseline characteristics of college students by level of depression.

Variable	Total	Normal	Mild depression	Moderate to severe depression	*p*
Age, year	24.0(22.0–26.0)	24.0(22.0–26.0)	24.0(22.0–26.0)	24.0(22.8–26.3)	0.687
Male, n(%)	168(47.7)	144(49.5)	17(48.6)	7(26.9)	0.087
Smoking status, n(%)					0.770
Non-smoker	338(96.0)	280(96.2)	34(97.1)	24(92.3)	
Former smoker	11(3.1)	8(2.7)	1(2.9)	2(7.7)	
Current smoker	3(0.9)	3(1.0)	0(0.0)	0(0.0)	
Drinking status, n(%)					0.351
Non-drinker	245(69.6)	205(70.4)	26(97.1)	14(92.3)	
Former drinker	8(70.4)	5(1.7)	0(2.9)	3(7.7)	
Current drinker	99(70.4)	81(1.0)	9(0.0)	9(0.0)	
BMI, kg/m^2^	21.4(19.7–23.1)	21.4(19.9–23.2)	21.0(18.9–22.7)	20.2(18.8–21.7)	**0.033**
Physical activity, n(%)					**0.004**
Low	66(18.8)	48(16.5)	6(17.1)	12(46.2)	
Moderate	196(55.7)	163(56.0)	22(62.9)	111(42.3)	
High	90(25.6)	80(27.5)	7(20.0)	3(11.5)	
Sleep quality, score	8.0(6.0–10.0)	7.0(6.0–9.0)	9.0(8.0–10.0)	9.0(7.0–12.0)	**<0.001**
Anxiety symptoms, n(%)					**<0.001**
Yes	267(76.9)	209(71.8)	35(100.0)	23(88.5)	

BMI, body mass index. Continuous variables are expressed as medians and 25th and 75th percentiles, and categorical variables are expressed as frequencies and percentages, *n* (%). Kruskal-Wallis tests, chi-square tests or Fisher exact tests were used for comparisons of baseline characteristics among the different levels of depression. Bold values indicated the results were statistically significant, with the two-tailed tests with *p*-values <0.05.

The results of the participants’ lung function assessments are reported in Table 2. The 352 participants had an average FEV1 of 3.8, and those with moderate-to-severe depression had lower PEF (*p* = 0.008), FVC (*p* = 0.005) and FEV1 (*p* = 0.006) values. The scatter plot of the correlation between the total SDS score and lung function stratified by sex, is presented in Supplementary Figures 1–6. A scatter plot shows a negative correlation between FEV1 values and the total SDS score among females (*p* = 0.041).

**Table 2:** Lung function of college students by level of depression.

Variable	Total	Normal	Mild depression	Moderate to severe depression	*p*
FEV1, L	3.6(3.2–4.3)	3.7(3.3–4.3)	4.0(3.2–4.4)	3.2(3.1–3.7)	**0.005**
FEV1 pp, %	92.7(81.0–103.7)	93.4(80.9–104.0)	88.8(83.1–96.2)	91.1(78.0–106.6)	0.522
FVC, L	4.1(3.7–5.1)	4.2(3.7–5.1)	4.7(3.7–5.2)	3.7(3.5–4.2)	**0.006**
FVC pp, %	83.7(72.0–94.4)	83.7(72.1–94.8)	83.1(71.0–91.8)	86.4(69.5–96.0)	0.861
FEV1/FVC, %	83.8(83.1–84.5)	83.8(83.1–84.5)	83.8(82.9–84.4)	84.2(83.4–84.7)	0.264
FEV1/FVC pp, %	117.4(111.8–119.4)	111.7(117.4–119.4)	118.1(109.1–119.3)	116.5(113.1–119.0)	0.871
PEF, L/s	7.6(7.1–9.8)	7.8(7.2–9.9)	9.4(7.2–10.0)	7.1(6.9–8.0)	**0.008**
PEF pp, %	89.6(78.0–100.0)	90.0(79.1–100.8)	86.8(71.7–100.1)	82.8(71.7–95.4)	0.128

FEV1, forced expiratory volume in 1 s; FVC, forced vital capacity; PEF, peak expiratory flow; FEV1 pp, predicted percentage of the forced expiratory volume in 1 s; FVC pp, predicted percentage of the forced vital capacity; FEV1/FVC pp, predicted percentage of the FEV1/FVC ratio; PEF pp, predicted percentage of the peak expiratory flow. Continuous variables are expressed as medians and 25th and 75th percentiles. Kruskal-Wallis tests, chi-square tests or Fisher’s exact tests were used for comparisons of baseline characteristics among the different levels of depression. Bold values indicated the results were statistically significant, with the two-tailed tests with *p*-values <0.05.

The association between the level of depression and lung function is shown in Table 3. In the unadjusted model, mild depression was associated with a significantly higher risk of a lower FEV1 (OR = 1.498, *p* = 0.003). After adjusting for sex and age in model 2, a significant relationship was found (OR = 1.290, *p* = 0.018). Similarly, in model 3, the association between mild depression and FEV1 remained relatively unchanged after adjusting further for various health-related factors (OR = 1.199, *p* = 0.044). On the other hand, moderate-to-severe depression was significantly but negatively related to FEV1 in the unadjusted model (OR = 3.546, *p* = 0.005) and model 2 (OR = 3.137, *p* = 0.020) and model 3 (OR = 2.980, *p* = 0.048). Furthermore, the unadjusted model indicated that mild depression was associated with a higher risk of a lower PEF (OR = 3.546, *p* = 0.008).

**Table 3:** Associations between level of depression and lung function among college students.

	FEV1		FEV1 pp		FVC		FVC pp	
Model 1								
	OR	95%CI	OR	95%CI	OR	95%CI	OR	95%CI
Normal	1.000	Reference	1.000	Reference	1.000	Reference	1.000	Reference
Mild depression	**1.498** †	**1.393–1.619**	1.596	0.781–3.260	0.893	0.527–1.514	1.229	0.608–2.484
Moderate and serious depression	**3.546** †	**1.384–9.086**	1.241	0.555–2.775	0.633	0.383–1.046	0.887	0.397–1.983
Model 2								
	OR	95%CI	OR	95%CI	OR	95%CI	OR	95%CI
Normal	1.000	Reference	1.000	Reference	1.000	Reference	1.000	Reference
Mild depression	**1.290** *	**1.156–1.570**	1.587	0.773–3.255	0.507	0.128–2.002	1.264	0.623–2.564
Moderate and serious depression	**3.137** *	**1.848–20.172**	1.147	0.509–2.584	2.589	0.491–13.666	0.853	0.378–1.922
Model 3								
	OR	95%CI	OR	95%CI	OR	95%CI	OR	95%CI
Normal	1.000	Reference	1.000	Reference	1.000	Reference	1.000	Reference
Mild depression	**1.199** *	**1.128–1.457**	1.332	0.626–2.834	0.428	0.101–1.807	1.236	0.586–2.608
Moderate and serious depression	**2.980** *	**1.767–21.832**	0.989	0.406–2.411	2.432	0.423–13.980	0.637	0.263–1.545
	FEV1/FVC		FEV1/FVC pp		PEF		PEF pp	
Model 1								
	OR	95%CI	OR	95%CI	OR	95%CI	OR	95%CI
Normal	1.000	Reference	1.000	Reference	1.000	Reference	1.00	Reference
Mild depression	0.951	0.471–1.918	0.872	0.431–1.761	0.798	0.393–1.619	1.317	0.651–2.661
Moderate and serious depression	0.448	0.189–1.062	1.207	0.540–2.700	**3.546** †	**1.314–9.011**	1.774	0.779–4.039
Model 2								
	OR	95%CI	OR	95%CI	OR	95%CI	OR	95%CI
Normal	1.000	Reference	1.000	Reference	1.000	Reference	1.00	Reference
Mild depression	1.276	0.392–4.160	0.820	0.400–1.680	0.495	0.156–1.565	1.222	0.595–2.511
Moderate and serious depression	0.860	0.230–3.210	1.062	0.468–2.408	4.103	1.846–19.895	1.632	0.704–3.781
Model 3								
	OR	95%CI	OR	95%CI	OR	95%CI	OR	95%CI
Normal	1.000	Reference	1.000	Reference	1.000	Reference	1.00	Reference
Mild depression	1.501	0.419–5.373	0.801	0.376–1.706	0.395	0.116–1.345	0.964	0.449–2.070
Moderate and serious depression	1.381	0.297–6.415	1.186	0.494–2.849	3.558	0.677–18.693	1.426	0.571–3.559

FEV1, forced expiratory volume in 1 s; FEV1 pp, predicted percentage of the forced expiratory volume in 1 s; FVC, forced vital capacity; FVC pp, predicted percentage of the forced vital capacity. The indicators of lung function and depression severity were used as dependent and independent variables, respectively. Multiple logistic regression models were used to explore the associations between level of depression and lung function. Model 1 is crude; Model 2 controls for age and sex; Model 3 controls for age, sex, BMI, smoking and drinking status, physical activity, anxiety symptoms and sleep quality. *p < 0.05, ^†^*p* < 0.01, ^‡^*p* < 0.001; exact p-values are reported in the Results section. FEV1/FVC pp, predicted percentage of the FEV1/FVC ratio; PEF, peak expiratory flow; PEF pp, predicted percentage of the peak expiratory flow. The indicators of lung function and depression severity were used as dependent and independent variables, respectively. Multiple logistic regression models were used to explore the associations between level of depression and lung function. Model 1 is crude; Model 2 controls for age and sex; Model 3 controls for age, sex, BMI, smoking and drinking status, physical activity, anxiety symptoms and sleep quality. *p < 0.05, ^†^*p* < 0.01, ^‡^*p* < 0.001; exact *p*-values are reported in the Results section. Bold values indicated the results were statistically significant, with the two-tailed tests with *p*-values <0.05.

The results of the sensitivity analysis are presented in Supplementary Table 1. Compared with the low tertiles of the SDS score, the medium tertiles were associated with lower FEV1 in the unadjusted and adjusted models (OR = 1.809, *p* = 0.002 and OR = 1.533, *p* = 0.018; OR = 1.405, *p* = 0.046, respectively). Significant negative associations were found between the high tertiles of the SDS score and FEV1 value in the unadjusted (OR = 2.156, *p* = 0.001) and adjusted models (*β* = 2.359, *p* = 0.020; OR = 2.300, *p* = 0.048).

## 4. Discussion

This study presents the results of a study on the association of depression with lung function in young adults. Of the 352 enrolled participants, 35 (9.9%) had mild depression and 26 (7.4%) had moderate-to-severe depression. Multiple linear regression analysis was conducted to explore the relationship between the two variables. After controlling for the effects of potential confounders, mild depression was found to be associated with reduced FEV1, and moderate-to-severe depression was associated with reduced FEV1. Moreover, the unadjusted model indicated that the mild depression was associated with a higher risk of a lower PEF. A sensitivity analysis indicated that a higher SDS score was independently correlated with decreased FEV1. However, additional studies are needed to support these results.

These findings confirm the association between depression and decreased lung function. Similarly, Ghaemi *et al*. found that depression was associated with medicated chronic pulmonary disease (asthma or COPD), and a meta-analysis reported that patients with COPD were twice as likely to have depressive symptoms compared with controls (12). However, although various studies have reported such associations between depression and respiratory diseases (13, 14), similar relationships among young adults have not been well researched. Guo et al. noted that severe depression was independently related to a decline in FVC among Chinese college students, but that study did not include measures of other lung functions, such as FEVI/FVC, FEV1 and PEF, among others (7).

The exact mechanism by which depression has debilitating effects on lung function remains unclear, but this could result from an immune response. It is common knowledge that depression can enhance the production of pro-inflammatory cytokines (15, 16). The excessive production of these compounds can result in abnormal endothelial function and impaired lung alveoli, making them jointly responsible for a subsequent decline in lung function (17). One of the characteristics of depression is the activation of oxygen and nitrogen species pathways, which may be responsible for DNA damage, protein oxidation and lipid peroxidation (18). Through these processes, oxidative stress plays an essential role in the development and progression of pulmonary dysfunction (18). The Autonomic Dysregulation Model, proposed by Miller, may explain how depressed emotional states lead to reduced lung function. The premise of this model is that depression is accompanied by a pattern of autonomic system dysregulation (specifically a cholinergic or vagal bias), with potential airway instability and compromise (19). Moreover, depression could be related to a decrease in physical activity, thereby contributing to a decline in lung function (4). Finally, these findings could also be related to unhealthy dietary behaviors. Individuals with depression are more likely to consume energy-dense foods than their healthy counterparts are. Such consumption may lead to systemic inflammation (20), which directly activates an innate immune response through the activity of the toll-like receptor 4 and circulating free fatty acids. This activity may increase interleukin-6 (IL-6), a pro-inflammatory cytokine that strongly induces neutrophil responses leading to airflow obstruction (21, 22) and reduced lung function. Overall, the correlation between depression and lung function offers new insights into the role of depression in reduced lung function. Further studies should confirm the possible association between depression severity and lung function in young adults.

This study has limitations. First, a causal relationship between depression and lung function cannot be assessed because of the study’s cross-sectional design. Second, the sample was limited to Beijing college students; therefore, our results may not represent college students from other regions of China. Third, pro-inflammatory cytokines were not assessed. Whether these factors mediated the link between severe depression and lung function in our study population is unknown. Fourth, depression might not have been accurately diagnosed; therefore, the two cut-off points (SDS scores of 40 and 45), which were used to distinguish between participants having mild and moderate-to-severe depression might not have been accurate. Finally, scatter plots of correlations between the total SDS score and lung function after stratification by sex showed a negative correlation between the FEV1 and total SDS score of females. Although the associations between the SDS score and indicators of lung function were not statistically significant among males, their scatter plots showed a tendency toward a negative relationship, which may be due to the relatively small sample size after stratification by sex. Hence, additional studies with large sample sizes are needed to confirm these results.

To conclude, the present study showed that college students from China with depression were more likely to exhibit reduced FEV1. However, causality should be assessed in future studies using prospective cohorts or interventional studies.

## Data availability statement

The original contributions presented in the study are included in the article/Supplementary material, further inquiries can be directed to the corresponding author.

## Ethics statement

The studies involving human participants were reviewed and approved by the Institutional Review Board of the Peking University Health Science Center (IRB00001052-22073). The patients/participants provided their written informed consent to participate in this study.

## Author contributions

SS and CW conceptualized the study. CW collected the data and wrote the manuscript. CW and HC analyzed the data. All authors read and approved the final manuscript.

## Funding

This study was supported by the National Key Research and Development Program of China (Nos. 2020YFC2008801 and 2020YFC2008800) and National Natural Science Foundation of China (No. 81972158).

## Conflict of interest

The authors declare that the research was conducted in the absence of any commercial or financial relationships that could be construed as a potential conflict of interest.

## Publisher’s note

All claims expressed in this article are solely those of the authors and do not necessarily represent those of their affiliated organizations, or those of the publisher, the editors and the reviewers. Any product that may be evaluated in this article, or claim that may be made by its manufacturer, is not guaranteed or endorsed by the publisher.
