# Supplementary material for: Association between depression and lung function in college students
Source: Front Public Health. 2023 Mar 23;11:1093935. doi: 10.3389/fpubh.2023.1093935 (PMC10078350; doi:10.3389/fpubh.2023.1093935)
Supplement: Supplementary file 1 [file Table_1.doc]

# Supplementary Table

**Supplementary Table 1** Associations between the tertiles of the SDS score and lung function among college students.

|  | FEV1 | | FEV1 pp | | FVC | | FVC pp | |
| --- | --- | --- | --- | --- | --- | --- | --- | --- |
| Model 1 | | | | | | | | |
|  | *OR* | *95%CI* | *OR* | *95%CI* | *OR* | *95%CI* | *OR* | *95%CI* |
| Low tertiles | 1.000 | reference | 1.000 | reference | 1.000 | reference | 1.000 | reference |
| Middle tertiles | **1.809**† | **1.379-2.365** | 0.826 | 0.490-1.394 | 0.869 | 0.515-1.466 | 1.004 | 0.596-1.694 |
| High tertiles | **2.156**† | **2.083-2.298** | 0.850 | 0.517-1.400 | 0.678 | 0.411-1.119 | 1.175 | 0.714-1.934 |
| Model 2 | | | | | | | | |
|  | OR | 95%CI | OR | 95%CI | OR | 95%CI | OR | 95%CI |
| Low tertiles | 1.000 | reference | 1.000 | reference | 1.000 | reference | 1.000 | reference |
| Middle tertiles | **1.533*** | **1.210-2.353** | 0.829 | 0.490-1.403 | 0.667 | 0.265-1.676 | 0.991 | 0.586-1.674 |
| High tertiles | **2.359*** | **2.242-2.542** | 0.881 | 0.532-1.458 | 0.658 | 0.272-1.593 | 1.158 | 0.700-1.915 |
| Model 3 | | | | | | | | |
|  | OR | 95%CI | OR | 95%CI | OR | 95%CI | OR | 95%CI |
| Low tertiles | 1.000 | reference | 1.000 | reference | 1.00 | reference | 1.000 | reference |
| Middle tertiles | **1.405*** | **1.207-2.457** | 0.905 | 0.521-1.575 | 0.686 | 0.261-1.803 | 1.060 | 0.611-1.839 |
| High tertiles | **2.300*** | **2.202-2.735** | 1.007 | 0.546-1.860 | 0.697 | 0.243-1.998 | 1.120 | 0.609-2.060 |

FEV1, forced expiratory volume in 1 second; FEV1 pp, predicted percentage of the forced expiratory volume in 1 second; FVC, forced vital capacity; FVC pp, predicted percentage of the forced vital capacity.

Multiple logistic regression models were used to explore the associations between tertiles of SDS score and lung function.

Model 1 is crude; Model 2 controls for age and sex; Model 3 controls for age, sex, BMI, smoking and drinking status, physical activity, anxiety symptoms and sleep quality.

The cut-off values of SDS scores tertiles were 27, 34.

* *p* < .05, † *p* < .01, ‡ *p* < .001; exact *p*-values are reported in the Results section.

**Supplementary Table 1** Associations between the tertiles of the SDS score and lung function among college students (continued).

|  | FEV1/FVC | | FEV1/FVC pp | | PEF | | PEF pp | |
| --- | --- | --- | --- | --- | --- | --- | --- | --- |
| Model 1 |  |  |  |  |  |  |  |  |
|  | *OR* | *95%CI* | *OR* | *95%CI* | *OR* | *95%CI* | *OR* | *95%CI* |
| Low tertiles | 1.000 | reference | 1.000 | reference | 1.000 | reference | 1.000 | reference |
| Middle tertiles | 0.992 | 0.588-1.676 | 1.141 | 0.676-1.924 | 0.933 | 0.533-1.574 | 0.685 | 0.405-1.158 |
| High tertiles | 1.254 | 0.762-2.065 | 0.852 | 0.517-1.403 | 0.701 | 0.425-1.155 | 0.823 | 0.500-1.355 |
| Model 2 |  |  |  |  |  |  |  |  |
|  | *OR* | *95%CI* | *OR* | *95%CI* | *OR* | *95%CI* | *OR* | *95%CI* |
| Low tertiles | 1.000 | reference | 1.000 | reference | 1.000 | reference | 1.000 | reference |
| Middle tertiles | 0.828 | 0.343-1.994 | 1.180 | 0.693-2.010 | 0.833 | 0.333-2.083 | 0.704 | 0.412-1.206 |
| High tertiles | 0.779 | 0.331-1.834 | 0.937 | 0.563-1.562 | 0.735 | 0.306-1.766 | 0.930 | 0.556-1.555 |
| Model 3 |  |  |  |  |  |  |  |  |
|  | *OR* | *95%CI* | *OR* | *95%CI* | *OR* | *95%CI* | *OR* | *95%CI* |
| Low tertiles | 1.000 | reference | 1.000 | reference | 1.00 | reference | 1.000 | reference |
| Middle tertiles | 0.697 | 0.269-1.809 | 1.168 | 0.671-2.034 | 0.923 | 0.353-2.418 | 0.831 | 0.473-1.461 |
| High tertiles | 0.592 | 0.197-1.778 | 1.020 | 0.547-1.901 | 0.827 | 0.292-2.346 | 1.223 | 0.651-2.296 |

FEV1/FVC pp, predicted percentage of the FEV1/FVC ratio; PEF, peak expiratory flow; PEF pp, predicted percentage of the peak expiratory flow.

Multiple logistic regression models were used to explore the associations between tertiles of SDS score and lung function.

Model 1 is crude; Model 2 controls for age and sex; Model 3 controls for age, sex, BMI, smoking and drinking status, physical activity, anxiety symptoms and sleep quality.

The cut-off values of SDS scores tertiles were 27, 34.

* *p* < .05, † *p* < .01, ‡ *p* < .001; exact *p*-values are reported in the Results sections.
