# Supplementary material for: Association between depression and lung function in college students
Source: Front Public Health. 2023 Mar 23;11:1093935. doi: 10.3389/fpubh.2023.1093935 (PMC10078350; doi:10.3389/fpubh.2023.1093935)
Supplement: Supplementary file 2 [file Data_Sheet_1.doc]

# Supplementary Figure Captions


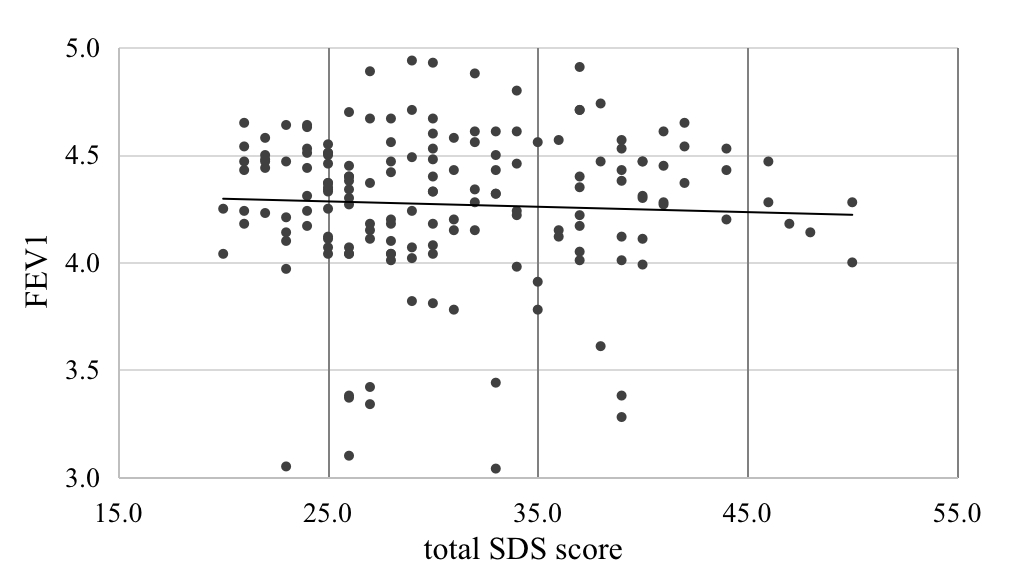


**Sup Fig. 1** Scatter plot of the correlation between SDS score and FEV1 among males


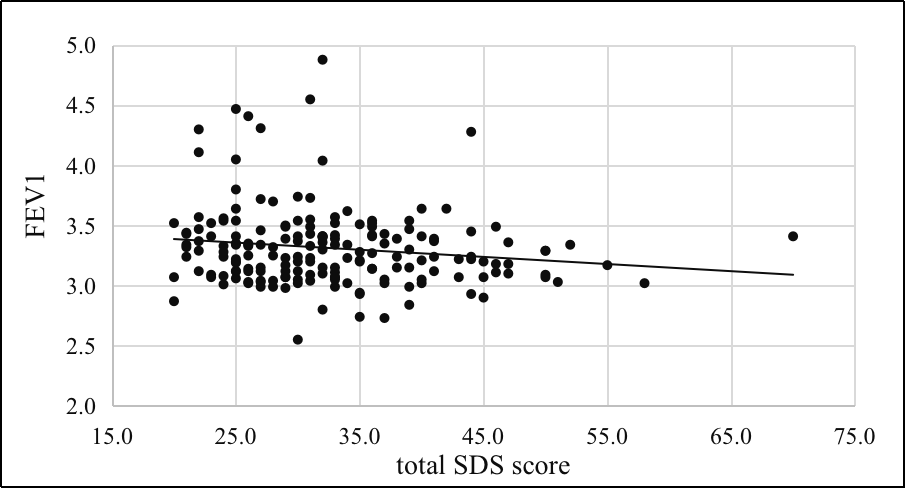


**Sup Fig. 2** Scatter plot of the correlation between SDS score and FEV1 among females


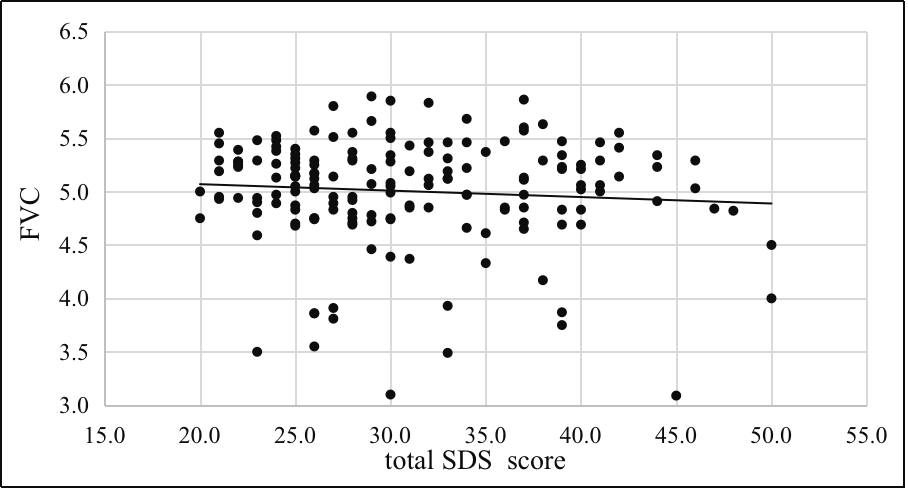


**Sup Fig. 3** Scatter plot of the correlation between SDS score and FVC among males


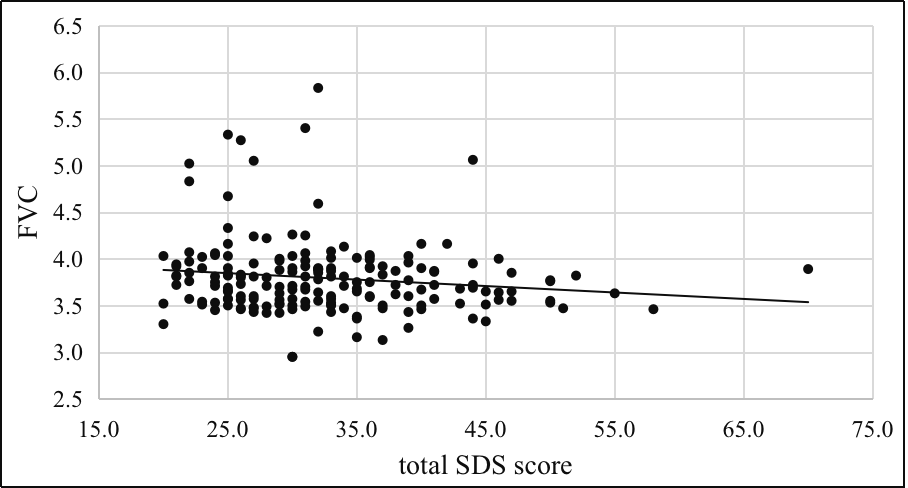


**Sup Fig. 4** Scatter plot of the correlation between SDS score and FVC among females


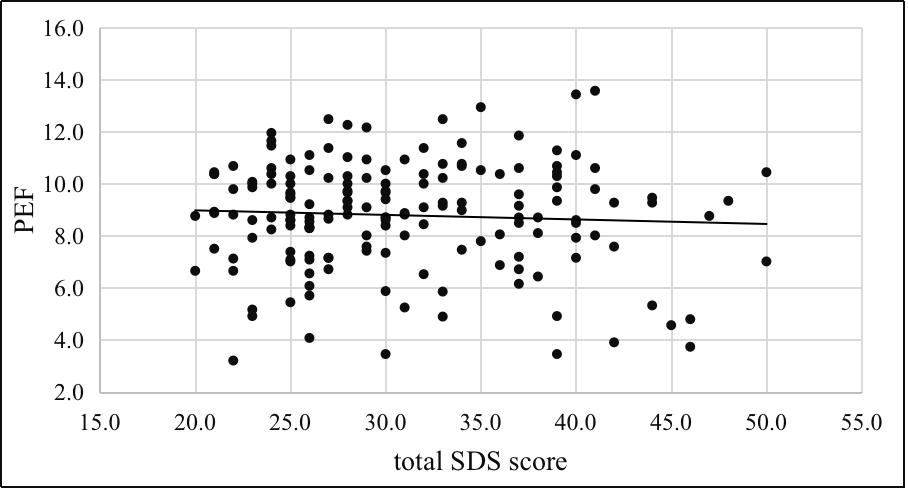


**Sup Fig. 5** Scatter plot of the correlation between SDS score and PEF among males


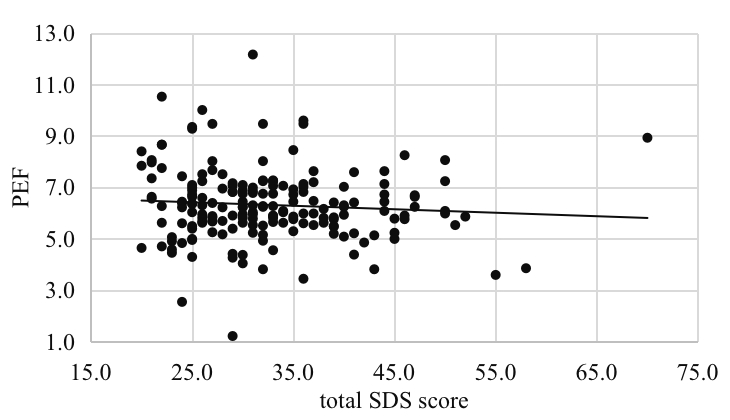


**Sup Fig. 6** Scatter plot of the correlation between SDS score and PEF among females
